# Severe liver disease resembling PSC in mice with K5-Cre mediated deletion of Krüppel-like factor 5 (Klf5)

**DOI:** 10.1007/s11248-021-00267-6

**Published:** 2021-06-11

**Authors:** Åsa Bergström, Marco Gerling, Noémi Van Hul, Carlos Fernández Moro, Björn Rozell, Rune Toftgård, Inderpreet Sur

**Affiliations:** 1 Department of Biosciences and Nutrition, Karolinska Institutet, 141 83 Huddinge, Sweden; 2 Tema Cancer, Karolinska University Hospital, 171 76 Stockholm, Sweden; 3 Department of Cell and Molecular Biology, Karolinska Institutet, 171 77 Stockholm, Sweden; 4 Department Clinical Pathology and Cancer Diagnostics, Karolinska University Hospital, 141 86 Stockholm, Sweden; 5 Department of Laboratory Medicine, Karolinska Institutet, 141 52 Huddinge, Sweden; 6 Department of Medical Biochemistry and Biophysics, Karolinska Institutet, 171 77 Stockholm, Sweden

**Keywords:** Knock-out mice, Animal model, PSC, Transcription factor, Liver

## Abstract

Chronic cholestatic liver diseases including primary sclerosing cholangitis (PSC) present a complex spectrum with regards to the cause, age of manifestation and histopathological features. Current treatment options are severely limited primarily due to a paucity of model systems mirroring the disease. Here, we describe the Keratin 5 (*K5*)*-Cre*; *Klf5*^*fl/fl*^ mouse that spontaneously develops severe liver disease during the postnatal period with features resembling PSC including a prominent ductular reaction, fibrotic obliteration of the bile ducts and secondary degeneration/necrosis of liver parenchyma. Over time, there is an expansion of Sox9^+^ hepatocytes in the damaged livers suggestive of a hepatocyte-mediated regenerative response. We conclude that Klf5 is required for the normal function of the hepatobiliary system and that the *K5-Cre*; *Klf5*^*fl/fl*^ mouse is an excellent model to probe the molecular events interlinking damage and regenerative response in the liver.

## Introduction

Krüppel-like factors are a family of transcription factors (TFs) that share highly conserved zinc-finger DNA-binding domains (Kaczynski et al. 2003). They show tissue-specific expression and have varied functions ranging from proliferation to differentiation. A striking function associated with some of the family members, including KLF5, is the ability to reprogram cell fates (Nakagawa et al. 2008). Although this function is best characterized with respect to induction of a pluripotent state, the role of Klf5 as a master transcription factor maintaining differentiation hierarchies in embryonic and adult tissues is also well documented (Fujii et al. 2012; Lin et al. 2010; Sur et al. 2006). As such Klf5 is also associated with the regenerative response following injury in tissues like the muscle and liver (Hayashi et al. 2016; Okada et al. 2018). In liver, Klf5 is highly expressed in the biliary epithelial cells while its expression is barely detectable in hepatocytes, that form the bulk of the liver mass. *Alfp-Cre* mediated liver-specific deletion of *Klf5* is well-tolerated in mice with no obvious phenotypic consequence unless mice are challenged with the hepatotoxin 3,5-diethoxycarbonyl-1,4-dihydrocollidine (DDC). Following DDC treatment, loss of Klf5 attenuates the ductular reaction (DR) characteristically observed after liver injury (Okada et al. 2018).

Following injury, liver can repair damaged tissue through proliferation of hepatocytes and cholangiocytes and by triggering fate switching between these two cell types. However, repeated cycles of damage and repair/regenerative response during chronic liver injury as seen for example in cholestatic liver diseases such as primary biliary cholangitis (PBC) and PSC, result in matrix deposition and progressive loss of liver function. The progression of the disease is particularly rapid in children. To identify new intervention points for treatment and drug validation, animal disease models are critical. In case of PSC, the *Mdr2*^*−/−*^ mouse model and mice fed with DDC have been useful for validation of therapeutic interventions and for obtaining mechanistic insight into disease progression (Fickert et al. 2006). However, while progress has been made, treatment options for these diseases are still limited and at the end often require liver transplantation. Generation of additional disease models is warranted to expedite research in the field.

In this study we demonstrate spontaneous development of cholestatic liver damage upon *K5-Cre* mediated *Klf5* deletion. The liver disease observed in these animals, replicates features associated with chronic cholestatic liver diseases in humans, in particular primary sclerosing cholangitis and vanishing bile duct syndromes. Our results suggest that *K5-Cre*; *Klf5*^*fl/fl*^ mice can be a valuable complement to existing mouse models of liver disease and further highlight the role of Klf5 for liver pathology.

## Results and discussion

**Fig. 1:**
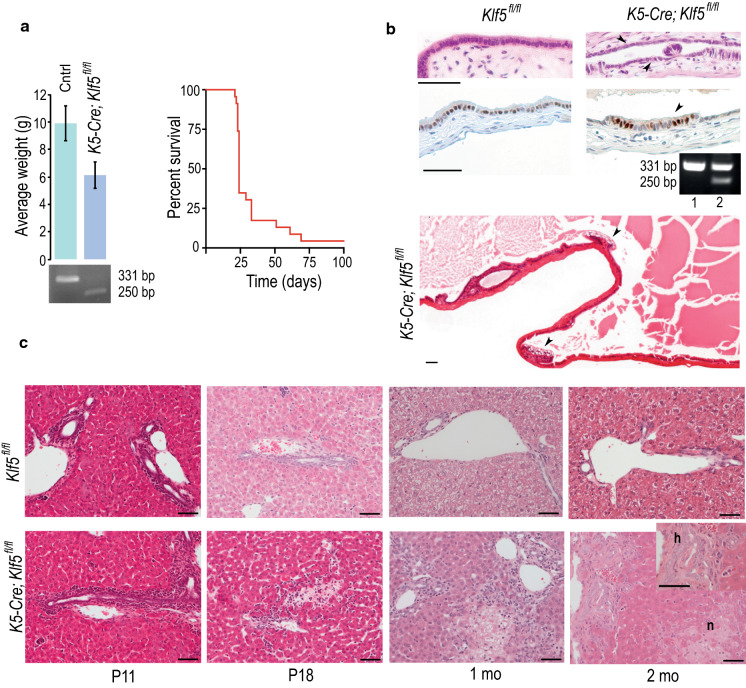
Compromised life span of *K5-Cre*; *Klf5*^*fl/fl*^ mice is associated with development of a severe liver disease in the postnatal period. **a** Left panel: *K5-Cre*; *Klf5*^*fl/fl*^ mice weigh less than the control mice at 3 weeks of age. Bottom: PCR analysis of genomic DNA isolated from primary keratinocytes in culture showing the targeted (331 bp) and floxed out (250 bp) alleles. Right panel: Kaplan–Meier survival plot showing compromised life span of *K5-Cre*; *Klf5*^*fl/fl*^ mice (n = 22). **b** Top panels: Hematoxylin–eosin (HE) stained sections of gallbladder epithelium. Arrow shows regions of flattened cuboidal cells appearing within the gallbladder epithelium of *K5-Cre*; *Klf5*^*fl/fl*^ mice. Middle panel: IHC showing restriction of nuclear Klf5 staining to the tall columnar epithelial cells (arrow) of the gallbladder. Inset: PCR analysis showing partial deletion of *Klf5* in *K5-Cre*; *Klf5*^*fl/fl*^ gallbladder (2) compared to control (1). Bottom panel: HE stained section of *K5-Cre*; *Klf5*^*fl/fl*^ gallbladder showing reactive/ degenerative changes in the epithelium (arrow). **c** HE stained liver sections showing ductular reaction, fibrosis (h) and necrotic foci (n) in the livers of *K5-Cre*; *Klf5*^*fl/fl*^ mice. All bars equal 50 μm

*Klf5*^*fl/fl*^ mice were crossed to *K5-Cre* mice that constitutively express Cre-recombinase from the bovine *K5* promoter that targets expression to the stratified epithelium including skin. Unlike the endogenous *K5* promoter, this transgene serendipitously also expresses Cre-recombinase in the gallbladder and biliary epithelium (Devos et al. 2017; Kiguchi et al. 2001). The *K5-Cre*; *Klf5*^*fl/fl*^ mice (*n* = 46) were born at the expected mendelian frequency and were indistinguishable from their wildtype littermates during the first week of birth. However, by two weeks of age the *K5-Cre*; *Klf5*^*fl/fl*^ mice were smaller and had developed a characteristic tail kink compared to littermate controls lacking either the Cre or the floxed alleles or both. Most of the mice had to be sacrificed around 4 weeks of age due to deteriorating general health, although a few animals survived until 2 months and one mouse till 10 months of age (Fig. 1a).

Necropsies of the *K5-Cre*; *Klf5*^*fl/fl*^ animals showed yellow coloration of the internal organs, a feature consistent with bile stasis, along with an enlarged gallbladder that was filled with bile. The gallbladder epithelium of the *K5-Cre*; *Klf5*^*fl/fl*^ mice had regions of flattened cuboidal cells interspersed between regions containing columnar cells (Fig. 1b) unlike the uniform layer of columnar cells with strong nuclear Klf5 expression that lined the wildtype gallbladder. Klf5 expression in the *K5-Cre*; *Klf5*^*fl/fl*^ mice was restricted to the columnar epithelial cells whereas the flattened cuboidal cells were completely devoid of it (Fig. 1b). Partial deletion of Klf5 in the gallbladder epithelium was also confirmed by PCR (Fig. 1b). In summary, loss of Klf5 resulted in a morphologically distinct cell type within the gallbladder epithelium. In addition, the gallbladder epithelium developed reactive and degenerative changes (Fig. 1b).

**Fig. 2:**
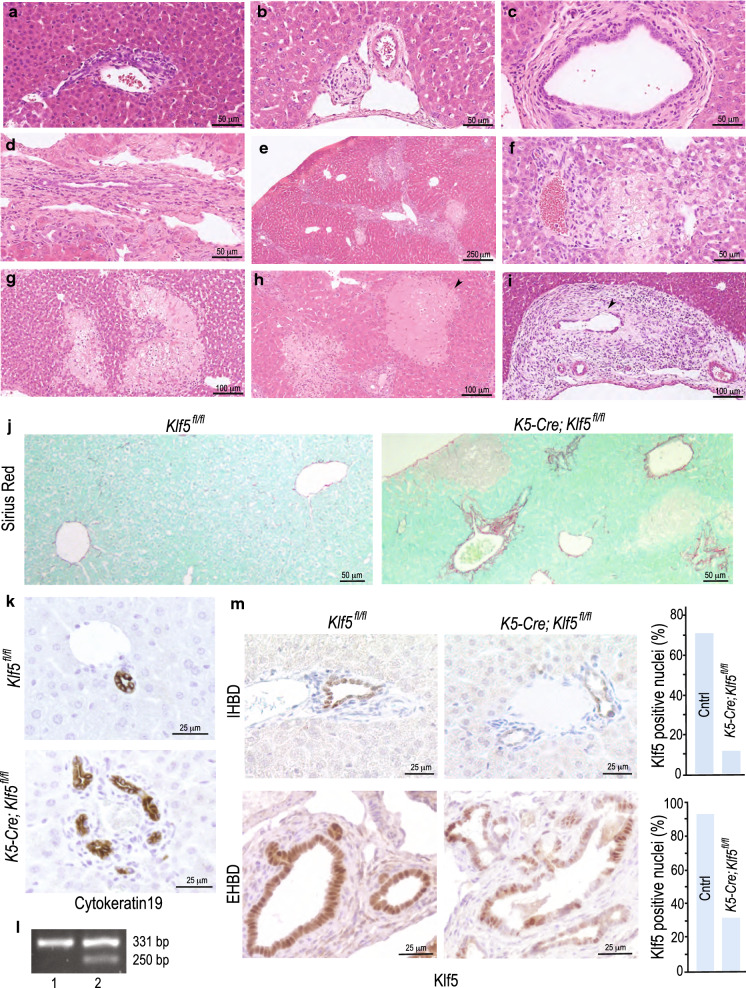
Liver lesions in *K5-Cre*; *Klf5*^*fl/fl*^ mice show striking similarity to those seen in humans with progressive chronic cholestatic diseases in particular PSC. **a** Small-sized portal zones showing ductular reaction where the original bile duct cannot be identified. **b** Degeneration of the biliary epithelium accompanied by occlusion of the lumen and incipient periductal fibrosis. **c** Large intrahepatic bile duct showing marked inflammation with numerous subepithelial and intraepithelial neutrophils and reactive/degenerative epithelial changes. **d** Large portal tract showing remnant of degenerated bile duct, portal fibrosis, mild inflammation and prominent ductular reaction. **e** Intensive portal fibrosis with bridging character. **f** The lobules showed patchy areas of hepatocyte damage, degeneration and necrosis consistent with cholestatic liver cell damage. **g** These progressed into larger areas of definite hepatocyte necrosis. **h** Part of the necrotic hepatocyte region presented coagulative character (arrow). **i** The extrahepatic bile ducts were also affected. The larger extrahepatic (perihilar) bile duct is shown with marked degenerative changes and partial loss of the epithelial lining (arrow). The surrounding periductal stroma shows intensive inflammation. **j** Sirius Red staining showing fibrotic lesions in *K5-Cre*; *Klf5*^*fl/fl*^ livers, **k** *K5-Cre*; *Klf5*^*fl/fl*^ livers exhibit ductular reaction. Bile ducts were detected with anti-cytokeratin19 antibody **l** PCR analysis demonstrating partial deletion of Klf5 in livers of *K5-Cre*; *Klf5*^*fl/fl*^ mice. Lane1: Klf5^fl/fl^, Lane2: *K5-Cre*; *Klf5*^*fl/fl*^. **m** Biliary epithelium of *K5-Cre*; *Klf5*^*fl/fl*^ mice has fewer cells with Klf5 in the nucleus. IHC showing expression of Klf5 in the biliary epithelial cells of intrahepatic (IHBD) and extrahepatic bile ducts (EHBD). Control bile ducts show prominent staining of the nucleus with anti-Klf5 antibody. In the *K5-Cre*; *Klf5*^*fl/fl*^ bile ducts, Klf5 expression is irregular. Histograms show percentage of Klf5 positive nuclei in the epithelium of IHBDs and EHBDs of control (Cntrl) and *K5-Cre*; *Klf5*^*fl/fl*^ mice. For IHBD (top panel)- 290 nuclei in control and 129 nuclei in *K5-Cre*; *Klf5*^*fl/fl*^ were counted. For EHBD (bottom panel)- 553 nuclei in control and 425 nuclei in *K5-Cre*; *Klf5*^*fl/fl*^ mice were counted

A detailed histological examination of the livers from mice aged between 11 days and 2 months revealed the progressive proliferation of intrahepatic bile ducts accompanied by peribiliary fibrosis, inflammation and multifocal hepatocyte necrosis (Fig. 1c). This pattern of lesions affected both the intrahepatic bile ducts of different sizes and the extrahepatic bile ducts (Fig. 2). The early biliary lesions were characterized by active portal inflammation with reactive and degenerative changes in the biliary epithelium followed by a prominent ductular reaction (Fig. 2a–d). The bile ducts subsequently underwent degeneration and fibrotic obliteration (Fig. 2b). Fibrotic portal expansion with bridging character developed in affected porta zones (Fig. 2e). Within 2 months of age, the lesion progressed with secondary degeneration and necrosis of numerous regions of the lobular liver parenchyma, suggestive of a cholestatic pathogenesis (Fig. 2f, h). The represented extrahepatic bile ducts (perihilar and peribiliary) also showed reactive and degenerative epithelial changes, periductal fibrosis and inflammation (Fig. 2i). Fibrotic lesions and hyperproliferation of the bile ducts were confirmed by Sirius Red and cytokeratin19 stainings (Fig. 2j, k). PCR analysis confirmed partial deletion of Klf5 within the livers of *K5-Cre*; *Klf5*^*fl/fl*^ mice (Fig. 2l). Since PCR reactions can have a bias towards specific product, we also performed IHC analysis of liver section using Klf5 antibody (Fig. 2m). Klf5 expression was detected in the epithelial cells lining both the intrahepatic bile ducts (IHBD) and the extrahepatic bile ducts (EHBD). Strongest expression was detected in mid to large sized IHBDs and EHBDs. In *K5-Cre*; *Klf5*^*fl/fl*^ mice, there was a drastic reduction in the number of biliary epithelial cells with nuclear Klf5 expression compared to controls (Fig. 2m). Only 12% of the IHBD epithelial cells in *K5-Cre*; *Klf5*^*fl/fl*^ livers expressed nuclear Klf5 compared to approx. 70% in the wildtype controls (Fig. 2m). In the control EHBDs almost all the cells lining the ducts (93%) had strong Klf5 expression while the *K5-Cre*; *Klf5*^*fl/fl*^ EHBDs exhibited a patchy regional Klf5 expression with approx. 68% of cells lacking Klf5 expression.

**Fig. 3:**
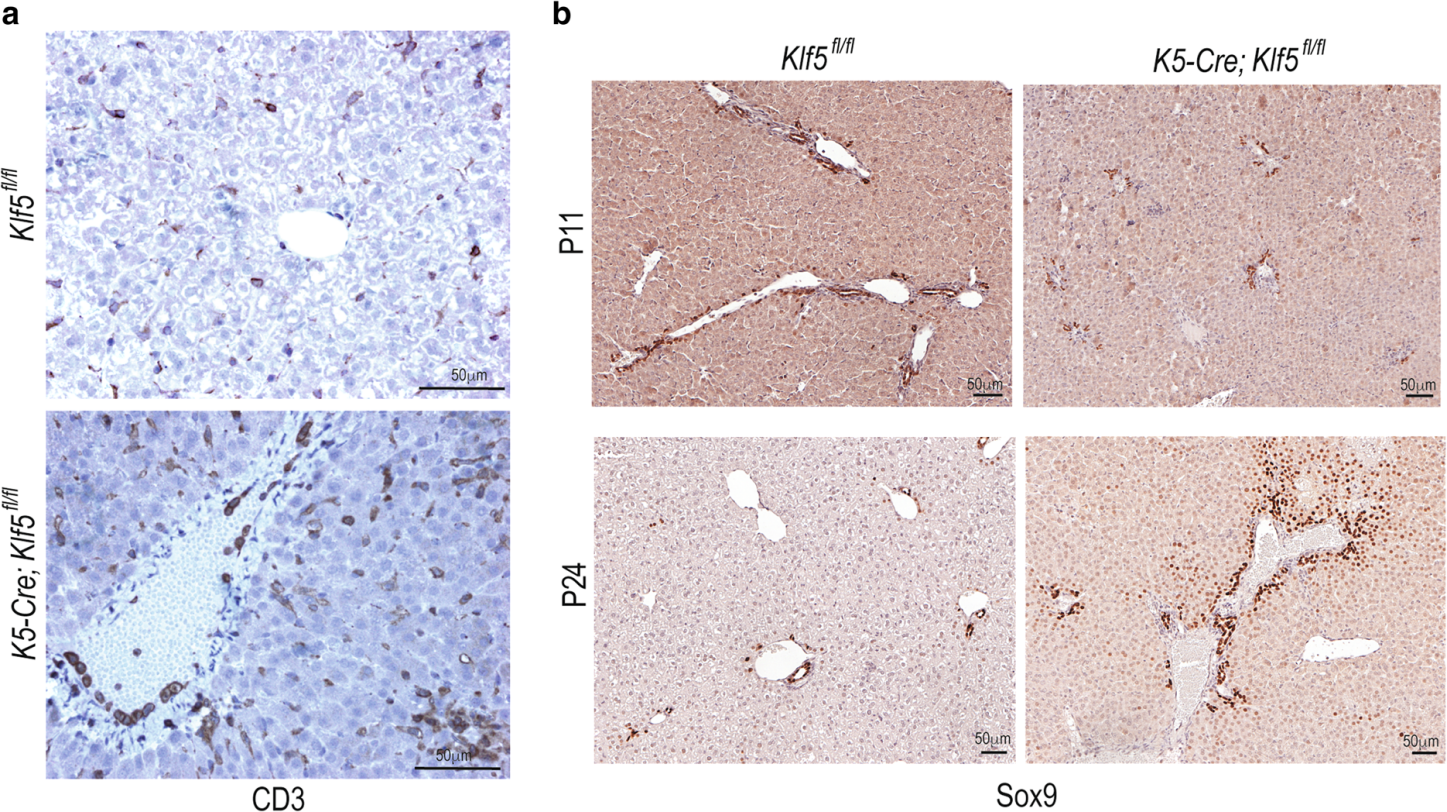
Infiltration of T-cells and transdifferentiation of hepatocytes to cholangiocyte lineage in *K5-Cre*; *Klf5*^*fl/fl*^ livers. **a** IHC analysis using anti-CD3 antibody showing active infiltration of T-cells into the liver parenchyma of *K5-Cre*; *Klf5*^*fl/fl*^ mice **b** Increased Sox9 expression in the livers of *K5-Cre*; *Klf5*^*fl/fl*^ mice at P11 (top) and p24 (bottom) showing transdifferentiation of hepatocytes into cholangiocyte lineage

We also detected an active infiltration of T-cells into the liver parenchyma of *K5-Cre*; *Klf5*^*fl/fl*^ mice (Fig. 3a) consistent with inflammatory changes in the liver. We did not detect inflammation or overt morphological changes in any of the other organs including the intestine. Concomitant with the disease progression, we observed a progressive increase in Sox9^+^ liver parenchymal cells. Although at P11 these cells were present close to the biliary tract, by 3–4 weeks there was an extensive expansion of the Sox9^+^ cells into the liver parenchyma indicating trans-differentiation of hepatocytes into cholangiocytes to repair the damage (Fig. 3b).

The pattern of liver damage observed in the *K5-Cre*; *Klf5*^*fl/fl*^ animals was consistent with a primary biliary affection with progressive degeneration of the bile ducts, followed by cholestatic liver damage which is accompanied by inflammation, ductular reaction and portal fibrosis. In humans, similar changes are seen in progressive chronic cholestatic diseases, in particular in PSC, but also in rare diseases such as vanishing bile duct syndrome in the context of drug-induced liver injury. In spite of a short life span the *K5-Cre*; *Klf5*^*fl/fl*^ mice provide an excellent opportunity to 1) molecularly dissect out events triggering a cholestatic damage induced repair response from the hepatocytes. 2) determine the critical cell types and signaling pathways involved in the process and 3) study aspects of neonatal cholestasis.

We conclude that Klf5 is required for the normal functioning of the intra and extra hepatic biliary tract, disruption of which results in cholestatic liver damage. Since the histological features observed in the *K5-Cre*; *Klf5*^*fl/fl*^ livers replicate changes seen in chronic cholestatic diseases, we propose *K5-Cre*; *Klf5*^*fl/fl*^ mice as a new genetic model for studying liver toxicity due to cholestatic injury. As such, this model complements existing mechanical and chemical models. Our results also emphasize the need to know the total expression patterns of *Cre* constructs, since an inadvertent activity may strongly influence the phenotype as in this case.

## Materials and methods

### Mice

Animal experiments were done in accordance with the regulations and with approval of the local ethical committee (Stockholm södra djurförsöksetiska nämnd). All mice were on a C57BL/6 background, maintained on a 12:12 h light cycle in standard plastic cages and provided with food and water ad libitum. Mouse lines and genotyping were as described before (Ramirez et al. 2004; Takeda et al. 2010).

### Immunohistochemistry (IHC)

IHC was performed on 4 μm FFPE sections with anti-Klf5 (1:100; kind gift from R. Nagai, Jichi Medical University), anti-Cytokeratin19 (1:200; Abcam), anti-CD3 (1:200; NovoCastra) and anti-Sox9 (1:1000; Millipore) antibodies. Nuclei of epithelial cells lining the intrahepatic bile ducts were counted for Klf5 expression in sections stained with anti-Klf5 antibody and counterstained with hematoxylin under the microscope.
